# Non-Cognitive Adaptive Resourcefulness: Scrutiny of Its Multidimensionality and Nomological Validity

**DOI:** 10.1177/00332941231221502

**Published:** 2023-12-15

**Authors:** Andrew Denovan, Neil Dagnall, Kenneth Drinkwater

**Affiliations:** Department of People and Performance, 5289 Manchester Metropolitan University, Manchester, UK; Department of Psychology, 5289 Manchester Metropolitan University, Manchester, UK

**Keywords:** Non-cognitive skills, mental toughness, ego resiliency, self-efficacy, perceived stress, anxiety control

## Abstract

Recent research has observed that Mental Toughness, Optimal Regulation, and Self-Efficacy share core features and variance. Investigators have named this commonality Non-Cognitive Adaptive Resourcefulness (NCAR). The NCAR validation study reported that the construct possesses promising psychometric properties, however, further research is required to replicate and extend these findings. Acknowledging this, the present paper using a UK-based sample of 1998 participants (*M*age = 40 years, range 18–83), tested the NCAR model against competing alternatives (multidimensional and one-factor solutions), and assessed the nomological validity of NCAR in relation to Perceived Stress and Anxiety Control. Participants completed the self-report study measures online. Exploratory structural equation modelling revealed that a bifactor solution represented data more effectively than one-factor and multi-factor alternatives. Additionally, a structural equation model found that NCAR significantly predicted Perceived Stress (subfactors of Distress and Coping) and Anxiety Control (subfactors of Emotional Control, Threat Control, and Stress Control). Moreover, NCAR predicted PSS Coping and Emotional Control to a greater extent than the specific bifactors (Mental Toughness, Optimal Regulation, and Self-Efficacy). This suggested that NCAR comprises an underpinning, positive psychological energy that facilitates coping. Particularly, an enabling resource that enhances the capacity to thrive under pressure and retain emotional control in demanding and trying circumstances.

## Introduction

The term non-cognitive skills refers to a broad range of capabilities (i.e., attitudes, mindsets, behaviours, and strategies) and constructs (e.g., mental toughness, grit, ego resiliency, self-efficacy, and hardiness), which sit outside the abilities assessed by formal aptitude tests (i.e., intelligence scales) (Ren et al., 2019). Although non-cognitive skills do not directly draw upon intellectual capacities, the label is misleading to the extent that non-cognitive skills implicitly draw on mental processes (Borghans et al., 2008). Theorists regard possession of non-cognitive skills as advantageous because they facilitate intrapersonal and interpersonal functioning. Commensurate with this supposition, researchers report that ownership of non-cognitive skills provides psychological benefits. For example, stress resistance and reduced depression (e.g., mental toughness, Mojtahedi et al., 2021; Papageorgiou et al., 2019, Perry et al., 2021; grit, Musumari, et al., 2018; resilience, Wu et al., 2013; and self-efficacy, Maciejewski et al., 2000). Consistent with these findings, evidence suggests that non-cognitive skills aid performance across a range of real-world settings (e.g., educational, Clough et al., 2016; occupational, Chakrabarti & Chatterjea, 2017; and sport, Dagnall et al., 2021).

Cognizance of the psychological benefits of non-cognitive skills has likely contributed to increased academic investigation in the area. This has identified, developed, and evaluated several conceptually distinct but overlapping constructs (e.g., hardiness, resilience, mental toughness, and grit). Despite the wealth of academic work, research in the area is conceptually limited because theorists tend to focus on constructs in isolation. This results in investigators either overlooking alternative non-cognitive skills or developing adversary positions (Fagioli et al., 2020). Noting this, Denovan et al. (2022a) advocated a complementary approach, contending that consideration of construct similarities would advance understanding by identifying the overlapping and additive properties of discrete non-cognitive skills.

Accordingly, in two independent studies, Denovan et al. (2022a) examined the commonality and distinctiveness of mental toughness, grit, ego resiliency, and self-efficacy. These non-cognitive constructs were selected because they were well established, had featured prominently in published research, and were often applied to real-world settings. The first study identified a general underlying non-cognitive factor. This was named Non-Cognitive Adaptive Resourcefulness (NCAR) and comprised Mental Toughness, Self-Efficacy, and the Optimal Regulation subscale of Ego Resiliency. The remaining non-cognitive elements, Grit and Openness to Life Experiences failed to load strongly on the factor. This suggested that these factors possessed unique features that contributed to construct uniqueness. The second study replicated study 1 outcomes and found that satisfactory invariance existed for both samples. Invariance in this context referred to model stability across the investigation. Hence, outcomes indicated that participants interpreted items consistently.

Examination of NCAR revealed that personal assurance and psychological adaptivity were core attributes. This aligned with the emphasis that Mental Toughness, Self-Efficacy, and Optimal Regulation place on belief in ability, confidence, and effective allocation of psychological resources. Collectively, these features suggest that NCAR is best delineated as a constructive mindset, which facilitates coping in negative and positive circumstances. This is conceptually important since some operationalisations of non-cognitive skills (i.e., resilience and hardiness) focus on adversity and coping (Denovan et al., 2022a), and fail to acknowledge that positive circumstances can also produce stress/pressure. Illustratively, in the context of sport, athletes routinely deal with anxieties related to both setbacks (i.e., injury and loss of form) and success (i.e., maintaining high levels of performance). In this context, the dynamic, changing nature of the environment results in the need to concurrently address multiple, diverse, fluctuating challenges. This broader definition of coping theoretically differentiates NCAR from non-cognitive skills that focus on the capacity to withstand and bounce back from hardship/difficulties.

This delimitation of NCAR was theoretically compelling as it subsumed generic definitions of the constitutional factors. In the case of mental toughness, this was possession of a flexible psychological resource that enables purposeful and effective performance/maintenance of goal-directed activities (Gucciardi, 2017). Regarding perceived self-efficacy, NCAR exemplified the idea that belief in one’s capabilities to produce designated levels of performance and influence life events was psychologically important (Bandura, 2006). In the case of optimal regulation, NCAR reflected the need to control psychological functioning in the face of stressors and crisis situations (Dębski et al., 2021). A further important feature of NCAR was flexibility and the ability to address changing situational demands. This interpretation aligned with core attributes of the contributing non-cognitive skills. Explicitly, the perception of mental toughness as a plastic, partially trainable trait, the emphasis self-efficacy places on environmental mastery, and the focus of ego-resiliency on effective psychological functioning (Farkas & Orosz, 2015).

The failure of Grit and the Ego-Resilience factor Openness to Life Experiences to contribute to NCAR reflected distinctiveness within these constructs. Illustratively both Perseverance of Effort (a dimension of Grit) and Openness to Life Experiences place an emphasis on skilled expressiveness and diligence (Klohnen, 1996). Indeed, Grit denotes perseverance and passion for long-term goals (Duckworth & Quinn, 2009). Explicitly the tendency to maintain interest over a sustained period. In contrast, inspection of NCAR parent scales (Mental Toughness, Self-Efficacy, and Optimal Regulation) revealed that flexibility, responsiveness, and environmental adaptability were prominent shared features.

### The Present Study

The first goal of this study was to assess the robustness of the NCAR model against competing alternatives. Specifically, a bifactor versus multidimensional conceptualisation. This was necessary because the validation study focused on bifactorial assessment. Hence, the analysis was necessary to determine whether NCAR was best represented by separate dimensions (multidimensional – consistent with previous theory), or as a unification of multi- and uni-dimensionality (bifactor).

The second intention was to test the nomological validity of the bifactorial structure in relation to Perceived Stress and Anxiety Control. Explicitly, to determine whether NCAR predicted these outcomes beyond Mental Toughness, Optimal Regulation, Self-efficacy. Previous research consistently links these constructs with coping ability, lower levels of stress, and management of anxiety (e.g., Dębski et al., 2021; Meyer et al., 2022; Nicholls et al., 2011; Schwarzer & Hallum, 2008). Since NCAR comprises additive, combined features of stress coping, resourcefulness, and general performance (inherent within the bifactors), it was expected that this factor would be a stronger predictor in comparison with the individual components. This research adopted a correlational approach for examining the objectives, focusing on regression-based predictive relationships alongside psychometric scrutiny.

## Method

### Sample

The sample consisted of 1998 UK-based participants, *M*age = 40 years, *SD* = 14.64, range 18–83. Specifically, 1442 females (72.17%), *M*age = 38.41 years, *SD* = 13.73, range 18–83; 541 males (27.83%), *M*age = 44.57 years, *SD* = 15.97, range 18–82; 10 non-binary (.50%), *M* = 27.40 years, *SD* = 7.94, range 18–46; and five who preferred not to disclose gender (.25%), *M* = 30 years, *SD* = 16.67, range 18–52. Recruitment was via Qualtrics survey platform. The exclusion criterion was that individuals must be at least 18 years of age. The researchers requested a representative sample from Qualtrics Audience Panel. Data collected via panels provides high quality data equivalent to traditional recruitment approaches (Kees et al., 2017). Qualtrics collates data from a pre-arranged pool of individuals consenting to take part in survey-based research. In this sense, the sample is opportunity-based.

### Measures

#### Mental Toughness

The 48-item Mental Toughness Questionnaire (MTQ-48) assesses the capacity to handle pressure and recover from setbacks via four primary factors: Control, Commitment, Challenge, and Confidence, (Clough et al., 2002). Due to the assessment of multiple constructs, the present study used the abridged, unidimensional, 10-item version (MTQ-10). The MTQ-10 was selected in preference to the original short version, the MTQ-18 (Clough et al., 2002) because it is psychometrically superior (Dagnall et al., 2019). Items within MTQ measures are presented as statements (e.g., “I generally feel in control”) and participants indicate their responses on a five-point Likert type scale (1 = Strongly Disagree to 5 = Strongly Agree). Higher scores indicate greater levels of mental toughness. Research has demonstrated robust psychometric properties for this scale (e.g., Dagnall et al., 2019; Denovan et al., 2021; Papageorgiou et al., 2018).

#### Optimal Regulation

The Optimal Regulation (OR) subscale from the Ego Resiliency Scale (ER89) (Block & Kremen, 1996) assessed qualities related to insight, confidence, and warmth, including the capacity to maintain homeostasis of the personality system in response to stressors/difficulties (Dębski et al., 2021). OR is a core feature of ego resiliency (Alessandri et al., 2012), alongside Openness to Life Experiences (i.e., productive activity and skilled Expressiveness) (see Alessandri et al., 2007). The scale presents items as statements (e.g., “I quickly get over and recover from being startled”) and participants indicate their level of endorsement via a four-point Likert type scale (i.e., 1 = Does Not Apply at All to 4 = Applies Very Strongly). Good psychometric properties exist for the OR subscale (Denovan et al., 2022b, 2022c).

#### Self-Efficacy

The 10-item General Self-Efficacy Scale (GSES) (Schwarzer & Jerusalem, 1995) measured participants’ belief in their ability to cope with life challenges and obtain desired outcomes. Within the GSES, items (e.g., “I am confident that I could deal efficiently with unexpected events”) appear as statements alongside a four-point Likert type response format (1 = “not at all true” to 4 = “exactly true”). The GSES is an established instrument, which possesses established psychometric integrity (Schwarzer & Jerusalem, 1995).

#### Perceived Stress

The 10-item Perceived Stress Scale (PSS-10) (Cohen & Williamson, 1988) assessed the degree to which participants appraised their current life as demanding and uncontrollable during the past month. Items are presented as statements (e.g., “In the past month, how often have you felt nervous and stressed”) and participants record their level of agreement on a five-point Likert type scale (0 = Never” to 4 = Very Often). Research has demonstrated that the PSS-10 includes two components: distress (PSS Distress) and ability to cope (PSS Coping) (Denovan et al., 2019). PSS Distress evaluates notions of feeling overwhelmed and upset, whereas PSS Coping indexes the extent to which an individual feels they can handle life problems/demands. Research provides psychometric support for this scale (e.g., Denovan et al., 2019).

Additional measures of perceived stress have been developed, with newer variants encompassing differing factors. For example, the Perceived Stress Inventory (Lee et al., 2015) includes Tension, Depression, and Anger dimensions. Thus, some inconsistency exists in relation to the mechanisms underpinning stress perception. However, the PSS-10 is a widely used and robust measure, receiving psychometric support in a variety of settings (Yilmaz Koğar & Koğar, 2023). Moreover, the PSS-10 captures stress appraisal consistent with a transactional approach, which is located within key early literature by Lazarus and colleagues (cf. Lazarus & Folkman, 1984).

#### Anxiety Control

The Anxiety Control Questionnaire (ACQ) evaluated perceived control related to external threats and emotional reactions (Rapee et al., 1996). The current study used the abridged version of the ACQ, which comprises 15-items (e.g., “I am able to control my level of anxiety”). This version, which is supported by preceding research (Brown et al., 2004), comprises three dimensions: Emotional Control, Threat Control, and Stress Control. Participants indicate responses using a five-point Likert type scale (1 = Strongly Disagree to 5 = Strongly Agree). The 15-tem ACQ has demonstrated good psychometric performance with both clinical and non-clinical participants (Brown et al., 2004).

### Procedure and Ethics

Prospective participants were sent a web-link accompanied by an information sheet, which described the study background, procedure, ethics details, and sought consent for taking part. Participants who consented advanced to the online survey where they were instructed to take their time and respond to all questions in an open and honest manner. Furthermore, participants were informed that questions measured preferences, and that no correct or incorrect responses existed. These instructions were implemented to limit socially desirable responding.

Study materials comprised an opening demographics section (i.e., age, preferred gender), measures, and a final section containing the debrief in which participants were reminded of the study’s purpose and their rights. To reduce potential order effects scale presentation rotated across participants. Ethical authorisation was awarded by the Manchester Metropolitan University Faculty of Health, Psychology and Social Care Ethics Committee (EthOS ID: 10,732).

### Analysis

Analysis progressed through two stages. The first replicated validation study outcomes. This was necessary to establish that the existence of a general non-cognitive factor comprised of Mental Toughness, Optimal Regulation, and Self-Efficacy was not an artefact of the validation study, and to demonstrate that the model generalised across samples. Correspondingly, analysis evaluated latent structure and compared models (i.e., multidimensional and bifactor solutions). For completeness, as a null test, a unidimensional model was considered.

Bifactor modelling enabled scrutiny of the degree of multidimensionality, whilst indicating the configuration and orientation of items loading on the general Non-Cognitive Adaptive Resourcefulness factor (Denovan et al., 2022b). In this context, a strength of bifactor modelling is the identification of systematic item variance relative to a general component and sources of supplementary variance, such as bifactors (Rodriguez et al., 2016a, 2016b).

Stage one analysis utilised exploratory structural equation modelling (ESEM). This assessed item effects across factors by not restricting non-target loadings to zero and permitting cross-loadings (Marsh et al., 2010). Target rotation was implemented. This involved allocating zero loadings to items that did not belong to the scale in relation to the model structure, while permitting other items to be free (Schonfeld et al., 2019).

The second stage of analysis involved testing the nomological validity of the supported factorial structure. This involved regressing the model onto two criteria measures, Perceived Stress (subfactors of PSS Distress and PSS Coping) and Anxiety Control (subfactors of Emotional Control, Threat Control, and Stress Control).

Throughout analyses, assessment of data-model fit used chi-square, Comparative Fit Index (CFI), Standardized Root-Mean-Square Residual (SRMR), and Root-Mean-Square Error of Approximation (RMSEA). Satisfactory fit includes CFI ≥.90, SRMR ≤.08 and RMSEA ≤.08 (Hu & Bentler, 1995). Bifactor model interpretation also employed the indices of Rodriguez et al. (2016a, 2016b). At the model level, this considered explained common variance (ECV), hierarchical omega (*ω*_*h*_), and percentage of uncontaminated correlations (PUC). Reise et al. (2013) concluded that PUC <.80, ECV >.60 and *ω*_*h*_ > .70 is suggestive of a robust general component, and a unidimensional model can be utilised in subsequent structural equation analyses without introducing more than 10% parameter bias.

Higher factor determinacy (FD >.90) and construct replicability (H >.80), in addition to stronger relative omega (*ω*) for the general versus bifactors, specified that a construct should be evaluated at a total score rather than bifactor level. Nonetheless, since this study was exploratory, it was not necessary to formulate sum scores among competing scales.

Factor loadings alongside item explained common variance (IECV) were considered at the item level. According to Stucky and Edelen (2015), IECV reflects the extent to which an item represents a general factor, with values >.5 implying greater weighting for a general rather than a specific bifactor (Winebrake et al., 2021). Correlations among latent factors were measured, using the criteria of Gignac and Szodorai (2016), i.e., .10, .20, and .30 represented small, typical, and large associations.

## Results

### Model Test

A one-factor ESEM model demonstrated unsatisfactory fit on CFI, χ^2^ (297) = 3312.66, *p* < .001, CFI = .86, RMSEA = .07 (.06, .07), SRMR = .05. Acceptable fit existed for the three-factor ESEM model, χ^2^ (248) = 1382.69, *p* < .001, CFI = .94, RMSEA = .04 (.04, .05), SRMR = .03. Assessment of the bifactor ESEM solution revealed satisfactory fit across indices, χ^2^ (225) = 1034.53, *p* < .001, CFI = .96, RMSEA = .04 (.04, .04), SRMR = .02. This was superior in comparison with the three-factor bifactor (AIC of 114,634.26 vs. 114,936.43). Bifactor specific criteria supported the existence of a general non-cognitive dimension underpinning the measures. Explicitly, the general factor possessed a *ω*_*h*_ of .84, ECV of .67, and PUC of .68. Moreover, FD of .95 and H of .93 existed. Satisfactory coefficient *ω* existed for the bifactors (Mental Toughness *ω* = .87, Optimal Regulation *ω =* .68, Self-efficacy *ω =* .91). However, relative *ω* inferred that each bifactor possessed a fairly low quantity of variance independent of the general dimension (Mental Toughness = .05, Optimal Regulation = .40, Self-efficacy = .35).

Scrutiny of target-rotated standardized item loadings (Table 1) supported these observations. Mental Toughness exhibited greater loadings on the general versus specific bifactor (mean loading of .57 vs. .12), as did Optimal Regulation (mean loading of .39 vs. .32) and Self-efficacy (mean loading of .57 vs. .41). Moreover, 84.61% of items possessed IECV >.5, and IECV <.5 existed only for Mental Toughness items 2 and 7, and Optimal Regulation items 1 and 4.

**Table 1.** Bifactor ESEM Factor Loadings and Item Explained Common Variance (IECV).

Scale	Sub-Scale	Item	General Factor	Bifactor			IECV
				MTQ10	OR	GSES	
MTQ10		Even when under considerable pressure I usually remain calm	.68	−.11			.97
		I tend to worry about things well before they actually happen	.45	.76			.25
		I usually find it hard to summon enthusiasm for the tasks I have to do	.39	.22			.76
		I generally cope well with any problems that occur	.74	−.14			.96
		I generally feel that I am a worthwhile person	.66	−.17			.93
		“I just don’t know where to begin” is a feeling I usually have when presented with several things to do at once	.42	.28			.69
		When I make mistakes, I usually let it worry me for days after	.42	.75			.23
		I generally feel in control	.68	−.07			.98
		I am generally able to react quickly when something unexpected happens	.62	−.18			.91
		I generally look on the bright side of life	.69	−.07			.98
ER89-R	OR	I get over my anger at someone reasonably quickly	.15		.35		.16
		My daily life is full of things that keep me interested	.55		.39		.66
		I usually think carefully about something before acting	.41		.21		.78
		Most of the people I meet are likable	.25		.43		.24
		I quickly get over and recover from being startled	.54		.23		.83
		I am generous with my friends	.45		.28		.71
GSES		I can always manage to solve difficult problems if I try hard enough	.51			.43	.57
		If someone opposes me, I can find the means and ways to get what I want	.35			.34	.50
		It is easy for me to stick to my aims and accomplish my goals	.54			.29	.77
		I am confident that I could deal efficiently with unexpected events	.67			.36	.76
		Thanks to my resourcefulness, I know how to handle unforeseen situations	.63			.43	.68
		I can solve most problems if I invest the necessary effort	.52			.51	.50
		I can remain calm when facing difficulties because I can rely on my coping abilities	.67			.35	.77
		When I am confronted with a problem, I can usually find several solutions	.58			.45	.62
		If I am in trouble, I can usually think of a solution	.57			.50	.56
		I can usually handle whatever comes my way	.63			.42	.68

*Note.* MTQ10 = mental toughness, ER89-R = ego resiliency, OR = optimal regulation, GSES = self-efficacy.

Collectively, these results supported the presence of a general non-cognitive dimension. Although, in comparison with the one-factor ESEM model, it appears that there is some support for the uniqueness of the specific bifactors. Otherwise, satisfactory fit would have occurred. Indeed, for Mental Toughness, reverse-keyed items (particularly 2 and 7) exhibited greater loadings on the bifactor. Furthermore, Optimal Regulation item 1 evidenced a particularly weak loading on the general factor (.15) and a loading of .35 on the bifactor. Controlling for these items resulted in a stronger ECV of .75 and weaker relative bifactor *ω* for Mental Toughness (.01) and Optimal Regulation (.33). Nonetheless, it was decided to retain these since they were core items within the standardised measures.

### Descriptive Statistics and Correlations

Skewness and kurtosis statistics were within the acceptable range of −2 to +2 (Byrne, 2010) (Table 2). Inspection of latent factor correlations demonstrated significant small to moderate associations among the bifactors. However, Mental Toughness correlated negatively with Optimal Regulation and Self-Efficacy in the context of the bifactor model. It is likely that this occurred due to the orientation of the bifactor once the variance had been accounted for by the general factor. Explicitly, the positively phrased items loaded strongly on the general factor, whereas the negatively keyed items took precedence on the bifactor. Indeed, consideration of latent factor associations in the three-factor ESEM solution reported large positive intercorrelations without the presence of the general factor (i.e., Mental Toughness with Optimal Regulation *r* of .37, Mental Toughness with Self-efficacy *r* of .62, Optimal Regulation with Self-efficacy *r* of .59).

**Table 2.** Descriptive Statistics and Correlations Among the Bifactors.

Variable	*M*	*SD*	Skew	Kurt	1	2	3
1. Mental toughness	31.19	6.67	−.21	.31		−.26**	−.33**
2. Optimal regulation	16.39	3.33	−.01	−.26	.37**		.18**
3. Self-efficacy	29.02	5.51	−.54	.81	.59**	.62**	

*Note.* Below the diagonal are associations from the correlated ESEM, above the diagonal are correlations from the bifactor ESEM; **p < .001.

### Nomological Validity

**Figure 1.**
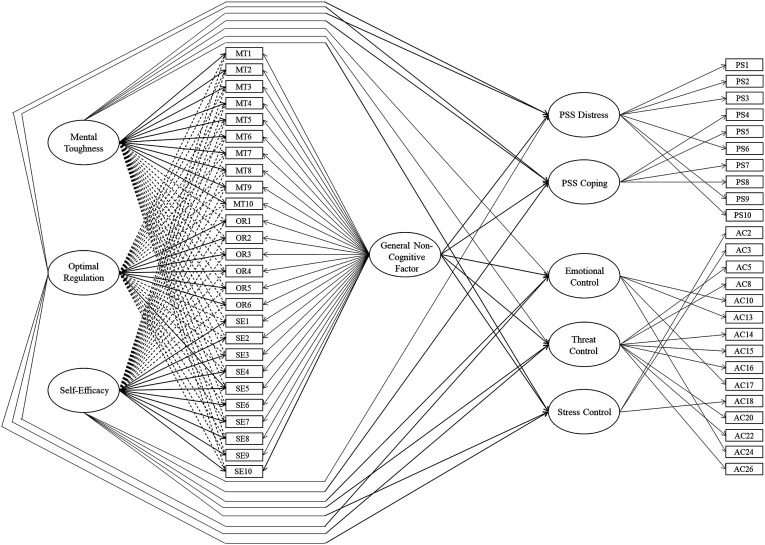
Schematic representation of the bifactor ESEM predictive model of perceived stress and anxiety control. *Note.* Full unidirectional arrows represent factor loadings and predictive relations, dotted unidirectional arrows represent cross-loadings.

A model (see Figure 1 for a schematic representation) contrasting the effects of the general factor and specific bifactors from the bifactor ESEM, in relation to criterion variables of Perceived Stress (comprising subfactors of PSS Distress and PSS Coping) and Anxiety Control (with subfactors of Emotional Control, Threat Control, and Stress Control), revealed satisfactory fit, χ^2^ (1120) = 5309.61, *p* < .001, CFI = .92, RMSEA = .04 (.04, .04), SRMR = .05. For Perceived Stress, the common variance among items captured by the general non-cognitive factor predicted PSS Distress negatively and significantly (*β* = −.36, *p* < .001). Optimal Regulation (*β* = −.09, *p* = .062) and Self-efficacy (*β* = −.03, *p* = .251) were not significant, whereas Mental Toughness was a stronger negative predictor of PSS Distress (*β* = −.68, *p* < .001). The general non-cognitive dimension was a stronger positive predictor of PSS Coping (*β* = .72, *p* < .001) than Mental Toughness (*β* = .21, *p* < .001) and Optimal Regulation (*β* = .10, *p* = .016), which were also significant positive predictors. Self-efficacy did not significantly predict PSS Coping (*β* = .02, *p* = .460).

In relation to Emotional Control, a similar pattern occurred to PSS Coping. Specifically, the general factor was a stronger positive predictor (*β* = .83, *p* < .001) than Mental Toughness (*β* = .20, *p* < .001) and Optimal Regulation (*β* = .25, *p* < .001). Moreover, Self-efficacy was not a significant predictor (*β* = .05, *p* = .112). Threat Control and Stress Control demonstrated similar relationships with the predictors, with Mental Toughness (*β* = .65, *p* < .001 and *β* = .71, *p* < .001) evidencing the strongest relationship, followed by the general dimension (*β* = .10, *p* = .002 and *β* = .34, *p* < .001). Optimal Regulation was not a significant predictor of Threat Control (*β* = .01, *p* = .940), yet significantly predicted Stress Control (*β* = .20, *p* < .001). Self-efficacy significantly predicted both constructs (*β* = .09, *p* = .002 and *β* = .12, *p* < .001).

These results indicated that the general non-cognitive dimension was a meaningful prognosticator of Perceived Stress and Anxiety Control, predicting constructs of PSS Coping and Emotional Control to a greater degree than the specific bifactors. Of the bifactors, only Mental Toughness related to the outcomes (PSS Distress, Threat Control, and Stress Control) more strongly than the general factor. The bifactor ESEM model predicted the outcomes substantially (PSS Distress *R*^2^ = .62, PSS Coping *R*^2^ = .56, Emotional Control *R*^2^ = .80, Threat Control *R*^2^ = .45, Stress Control *R*^2^ = .73), suggesting that the model possessed strong explanatory power.

## Discussion

Outcomes from the present investigation aligned with the validation study of Denovan et al. (2022a). Particularly, the finding that Mental Toughness, Optimal Regulation, and Self-Efficacy combined to form a general, Non-Cognitive Adaptive Resourcefulness (NCAR) factor. Additionally, results were consistent with the supposition that NCAR denotes high levels of self-reported stress-coping, management of problems/demands, environment adaptivity, and efficient performance. These conclusions were supported by the test of nomological validity, which found that NCAR significantly predicted Perceived Stress (subfactors of PSS Distress and PSS Coping) and Anxiety Control (subfactors of Emotional Control, Threat Control, and Stress Control). Specifically, NCAR predicted PSS Coping and Emotional Control to a greater extent than the bifactors.

This further suggested that the psychological underpinning of NCAR comprised coping ability, particularly the capacity to thrive under pressure and retain emotional control in demanding and trying circumstances. These findings imply that an additive feature of NCAR is solution-orientation, whereby individuals actively initiate and believe in their abilities to address challenges and obstacles. This interpretation concurs with Denovan et al. (2022a), who concluded that NCAR reflected belief in ability, confidence, and effective allocation of psychological resources. Since these inferences derive only from the validation and present investigation, subsequent studies tests should test them rigorously. This requires consideration of a broad range of appropriate convergent and discriminatory measures. For instance, it is conceptually important, given that hardiness possesses qualities such as adaptability and ability to withstand stress/demand (Caza et al., 2020), to determine the extent to which NCAR relates to this. Another construct to consider is trait Emotional Intelligence, which facilitates adaptive reactions to stress alongside quicker post-threat recovery (Lea et al., 2019).

Analyses revealed why stronger results for the general factor did not occur in the ESEM. Explicitly, reverse-keyed items for Mental Toughness and an unrelated Optimal Regulation item weakened ECV. This suggests that the general NCAR factor is underpinned by positive non-cognitive energy that does not link as strongly to negative features such as worry and absence of purpose, which are encapsulated within the reversed MT items (e.g., ‘I tend to worry about things well before they actually happen’ and ‘“I just don’t know where to begin” is a feeling I usually have when presented with several things to do at once’). This supposition fits with the weaker predictive relationships with Threat Control and Stress Control, which comprise reverse-keyed items indexing negative features allied to anxiety (e.g., ‘There is little I can do to change frightening events’).

In addition, the predictive model revealed that some bifactors retained unique predictive capacity relative to Perceived Stress and Anxiety Control (particularly Mental Toughness). Indeed, Mental Toughness demonstrated a stronger predictive relationship than NCAR. This potentially occurred because Optimal Regulation and Self-efficacy contain aspects that weaken the predictive capacity of NCAR relative to stress and anxiety control. Explicitly, Optimal Regulation relates to a general ability to stabilise psychological functioning, and to flexibly modify emotions, impulses, and reactions in association with the environment (Dębski et al., 2021; Farkas & Orosz, 2015). Moreover, the Self-efficacy measure that was utilised (GSES) captured general levels of self-belief (Schwarzer & Jerusalem, 1995), rather than self-efficacy specific to managing difficult situations (e.g., coping self-efficacy) (Chesney, 2006), whereas Mental Toughness possesses comparatively more stress/anxiety-specific features. This relates to the ability to adapt during a stressor to function at a high-level in the moment (Satterwhite, 2016). This greater degree of specificity (vs. a more general focus on self-belief and control of impulses), potentially explains why Mental Toughness was a stronger predictor.

### Limitations

A limitation of this study was the use of a cross-sectional design, which measured constructs at only one point in time. To establish the effectiveness of the proposed theoretical model, future studies should assess the stability and magnitude of this relationship over time using multiple time points. This will facilitate greater understanding of the emergent construct and ensure that NCAR consistently acts a psychological buffer against constantly changing life pressures. This is particularly important because stress and anxiety represent significant, enduring health concerns within society (Schneiderman et al., 2005).

Another limitation concerns the use of the MTQ-10 to assess mental toughness. The MTQ-10 is a brief, unidimensional measure, which was selected in preference to a longer, multidimensional scale (e.g., the MTQ48; Clough et al., 2002). This choice was informed by the fact that the test battery was lengthy due to the inclusion of multiple constructs. The general advantage of restricting survey length is higher completion, lower dropout, and reduced cognitive load on respondents. While the MTQ-10 has received considerable psychometric support (e.g., Dagnall et al., 2019), it assesses less domain content than longer measures and provides only a general index of mental toughness. This is potentially problematic since there are debates about the precise nature of mental toughness and the construct’s dimensionality. For example, the MTQ48 assesses four distinct but related dimensions (i.e., Challenge, Control, Commitment, and Confidence), which contribute to a general factor. These factors are important because they reflect and can influence performance in real-world settings in different ways (Drinkwater et al., 2019).

Since these dimensions could potentially interact with NCAR in different ways, the inclusion of the MTQ48 in subsequent investigations would conceptually enhance understanding of non-cognitive skills generally and provide specific insights into the function of NCAR. Lastly, analysis of the contribution of additional non-cognitive constructs (e.g., emotional intelligence and hardiness) to NCAR would have been useful, and more stress/anxiety-appropriate measures of self-efficacy could have been applied alongside the GSES (such as the coping self-efficacy scale). However, as this last point was arrived at post-hoc, this could be a compelling avenue for future research. These additional research efforts would provide further conceptual grounding for this new and emergent construct.

### Conclusion

This study supported the notion that core non-cognitive skills (i.e., Mental Toughness, Optimal Regulation, and Self-Efficacy) form a general domain. This is best conceptualised as NCAR, which is characterised by effective stress resistance, active management of problems/demands, situational flexibility, and efficient performance. The outcomes of the test of nomological validity (the construct’s relationship to theorized outcomes) were commensurate with this elucidation. Particularly, they were consistent with the notion that higher scores on NCAR denote the presence of a positive psychological mindset that manifests as active coping, the ability to maintain control in demanding circumstances, and the capacity to thrive under pressure. Since these conclusions derive from the present and validation studies, further work is required to ensure that findings generalise across samples and contexts (e.g., occupational, educational, sports settings). Similar to the development and assessment of mental toughness researchers could best achieve this through the use of theoretically appropriate concurrent and discriminatory measures and methodologies (i.e., case study, psychometric evaluation, and interview-based). Concomitantly, triangulation between quantitative and qualitative approaches would provide deeper understanding of the nature of NCAR from both general and personal perspectives.

## Data Availability

The dataset generated during and/or analysed during the current research is accessible via Figshare: https://doi.org/10.6084/m9.figshare.22770671 (Denovan et al., 2023).
